# A theory of rapid evolutionary change explaining the *de novo* appearance of megakaryocytes and platelets in mammals

**DOI:** 10.1242/jcs.260286

**Published:** 2022-12-14

**Authors:** John F. Martin, Pier Paolo D'Avino

**Affiliations:** ^1^ Division of Medicine, University College London, 5 University Street, London WC1E 6JF, UK; ^2^ Department of Pathology, University of Cambridge, Tennis Court Road, Cambridge, CB2 1QP, UK

**Keywords:** Cytokinesis, Evolution, Megakaryocytes, Platelets, Polyploidy

## Abstract

Platelets are found only in mammals. Uniquely, they have a log Gaussian volume distribution and are produced from megakaryocytes, large cells that have polyploid nuclei. In this Hypothesis, we propose that a possible explanation for the origin of megakaryocytes and platelets is that, ∼220 million years ago, an inheritable change occurred in a mammalian ancestor that caused the haemostatic cell line of the animal to become polyploid. This inheritable change occurred specifically in the genetic programme of the cell lineage from which the haemostatic cell originated and led, because of increase in cell size, to its fragmentation into cytoplasmic particles (platelets) in the pulmonary circulatory system, as found in modern mammals. We hypothesize that these fragments originating from the new large haemostatic polyploid cells proved to be more efficient at stopping bleeding, and, therefore, the progeny of this ancestor prospered through natural selection. We also propose experimental strategies that could provide evidence to support this hypothesis.

## Teaser

**Summary:** Platelets are unique to mammals and produced from large polyploid megakaryocytes. Here, we hypothesize that platelets might have evolved because of a single event that led to the megakaryocyte polyploidization in a mammalian ancestor.

## Introduction

Evolution through natural selection as described by Darwin is the bedrock of biodiversity (Darwin, 1859). Among modifications to this theory, Gould's concept of punctuated equilibrium in evolution postulated that exaptation (see Glossary) could occur, truncating the time needed for evolution (Eldredge and Gould, 1972). He gave the example of the cooling feathers of dinosaurs becoming the flight feathers of birds over a shorter time than if Darwinian gradualism were applied. These theories remain descriptive without controlled interventional experimental proof. We would like to propose that, in limited circumstances, biological diversity can occur very rapidly in a single animal that, along with its progeny, can then prosper through natural selection. Importantly, in this Hypothesis article, we also propose experimental approaches that can be undertaken to test the theory.

**Glossary**

**Demarcation membrane system (DMS):** The cytoplasm of the megakaryocyte is penetrated, uniquely among cells, by many invaginations of plasma membrane. These will become the future plasma membranes of platelets. The DMS contains plasma. In transmission electron microscopy, the DMS gives the megakaryocyte cytoplasm a ‘Swiss cheese’ appearance.

**Endomitosis:** A variation in the cell cycle where mitosis is initiated, but not completed. This will give rise to either a mononucleated polyploid cell if karyokinesis is impaired, or to a binucleated polyploid cell if cytokinesis is unsuccessful.

**Epigenetic:** Epigenetic changes are modifications to DNA that determine whether genes are turned on or off, thus controlling gene activity without changing DNA sequence.

**Exaptation:** A feature of an organism that takes on a function where none previously existed or where it differs from its previous function, which had been evolved. The word was first proposed as a replacement for ‘preadaptation’ in evolutionary theory.

**Inferior vena cava:** This the largest vein in the mammalian body. It sits at the posterior abdominal wall on the right side of the aorta, the largest artery in the body. It carries blood drained from the lower limbs, the pelvis and the abdomen. Its major function is to carry de-oxygenated blood to the heart from where the blood enters the circulation of the lung vessels where the blood is re-oxygenated.

**Log Gaussian:** Data follow a Gaussian or normal distribution when scatter or dispersion is caused by the sum of many independent and equally weighted factors. An example is the bell-shaped curve of height at a given age and given sex in the human population. When the dispersion is caused by the product (as opposed to the sum) of many and independent and equally weighted factors then data follow a log Gaussian or log normal distribution. When plotted on an *x*-axis the curve is skewed to the right with a tail. When the log Gaussian distribution is plotted on a logarithmic *x*-axis then a Gaussian, bell-shaped, curve is seen.

**Oblate spheroid:** The shape of a rugby football.

**Polyploidy:** The condition in which a normally diploid cell or organism acquires one or more additional sets of chromosomes.

All mammals have platelets, which are necessary for survival because they arrest bleeding (haemostasis). No other order has platelets, even though the problem of bleeding exists. A solution to the enigma of why only mammals have platelets was recently proposed, linking the origin of the platelet in an egg-laying animal, which initially did not have platelets, to the evolution of the eutherian (haemochorial) placenta found in all mammals apart from monotremes and marsupials (Martin and Wagner, 2019). In this paper, it was suggested that, without powerful haemostasis, the mammalian mother might die from bleeding when the placenta separates from the uterus, leaving the new-born without a source of nutrition. This problem would not occur in egg-laying animals whose young are immediately independent. Therefore, the origin of platelets in an egg-laying animal, possibly an ancestor of the modern duck billed platypus, a member of the monotreme order, which lays eggs and has platelets (Canfield and Whittington, 1983; Hughes and Hall, 1998), was causal in the evolution of mammals.

**Fig. 1.**
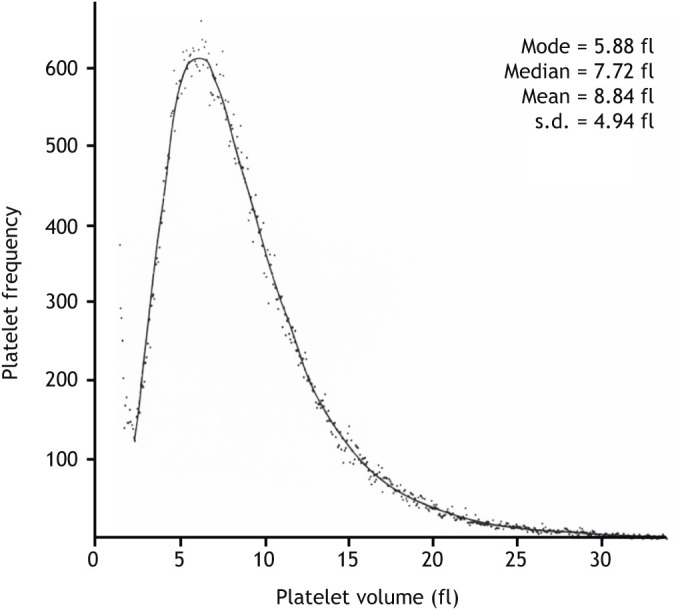
**Platelet volume distribution, unlike cell volume distribution of cells produced by mitosis, fits a log Gaussian curve.** Scatter diagram showing the frequency distribution of platelet volumes in a representative human platelet population, measured by volume displacement, using a Coulter counter. Values for mode, median, mean and standard deviation are shown. The continuous line indicates the predicted log Gaussian curve for these values. Reproduced from Martin et al. (2012).

**Fig. 2.**
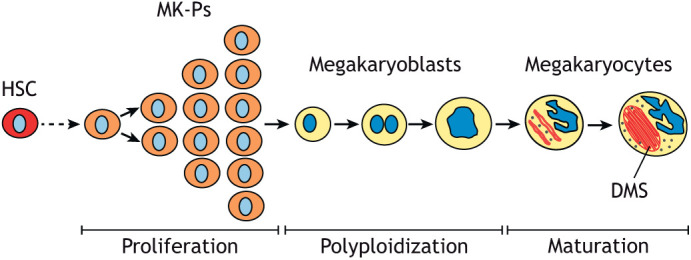
**Polyploidization precedes the final maturation steps during megakaryocyte differentiation.** Schematic diagram illustrating megakaryocyte differentiation. Megakaryocyte progenitors (MK-P) originating from an hematopoietic stem cell (HSC) proliferate through normal mitotic divisions until they start to become highly polyploid through a series of aborted cell divisions, or endomitoses, to produce megakaryoblasts. Note that the first tetraploid megakaryoblast contains two nuclei because we propose that it most likely originates via cytokinesis failure (see text for details). After polyploidization, the megakaryoblasts mature into megakaryocytes by increasing their cytoplasm through enhanced protein synthesis and by producing specific granules (grey dots) and a demarcation membrane system (DMS, in red).

**Fig. 3.**
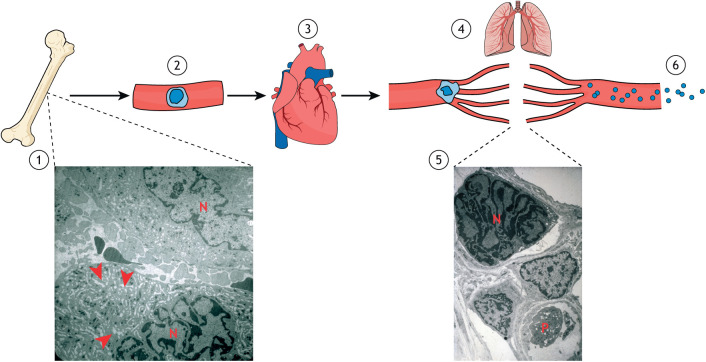
**The modern megakaryocyte recapitulates the route taken in its evolutionary origin.** The schematics illustrate the journey of the megakaryocyte from the bone marrow to the lungs. Having matured in the bone marrow, the megakaryocyte leaves and enters a vein draining the bone marrow (in our theory, we propose this exit to the venous system occurred in the premammalian thrombocytes with polyploid nuclei). This vein leads to the great vein at the back of the abdomen and thorax, the inferior vena cava (2), which is a low-pressure blood vessel that carries blood to the heart (3) where the blood first enters the right atrium and then the right ventricle. There it receives kinetic energy to enter the higher-pressure vessel, the pulmonary artery (4). The megakaryocyte in this high-pressure blood is propelled into the capillary bed of the pulmonary circulation where, partly because of the disparity in size, its cytoplasm fragments into platelets, aided by the local shear forces. The cytoplasmic particles resulting from megakaryocyte cytoplasmic fragmentation are the platelets that leave the pulmonary circulation via the pulmonary vein to enter the systemic circulation (6). The two electron micrographs (taken at 5000×) show tissues taken from the bone marrow (1) and lungs (5) of a rabbit that underwent 6 days of intravenous injection of anti-platelet serum to amplify megakaryocyte production in order to be able detect circulating megakaryocytes, which under normal platelet production are only found in low numbers in the circulation. In the bone marrow (1), the para sinusoidal membrane runs from the top left to mid right of the micrograph. This membrane separates the bone marrow from the venous system. At the top right, a megakaryocyte can be seen in the bone marrow, whereas at the bottom a megakaryocyte is in the venous system. Their nuclei are marked N and the arrowheads indicate the demarcation membrane system (DMS). In lung tissue from the same experiment (5), a naked megakaryocyte nucleus (marked as N) that has lost its cytoplasm can be observed in a capillary. A large platelet (marked as P), as well as two smaller ones, can be seen in the vessel. The ‘empty’ space at the top right is an air-filled alveolus. Electron micrographs reproduced with permission from Slater et al. (1983); copyright Elsevier.

Platelets are very small and uniquely are anucleate from their genesis (Li et al., 2017). They also have two unique properties: (1) they are produced from large polyploid (see Glossary) precursors, the megakaryocytes (Stalker et al., 2012), and (2), unlike other cells, they present a unique log Gaussian cell volume distribution (Fig. 1; also see Glossary) (Paulus, 1975). Mature megakaryocytes are some of the largest cells in the mammal outside of the central nervous system, and each megakaryocyte produces ∼3000 platelets (Trowbridge et al., 1982). Megakaryocytes are among the few cell types in mammals that are highly polyploid; they can have up to 128N DNA content, with a modal ploidy of 16N in normal equilibrium in humans (Penington and Olsen, 1970). It is well known that an increase in ploidy is associated with an increase in cell size (Schoenfelder and Fox, 2015). Megakaryocytes originate in the bone marrow from hematopoietic stem cells (HSCs), which can commit toward different differentiation processes, including one that leads to megakaryocyte-committed progenitors (Choudry et al., 2021). These progenitors, called promegakaryoblasts or megakaryocyte precursors (MK-Ps), proliferate through normal mitotic divisions and begin to produce factors important for platelet function (Noetzli et al., 2019). At one point during the differentiation process, megakaryocyte precursors become highly polyploid through a series of aborted cell divisions, or endomitoses (see Glossary) (Mazzi et al., 2018). After polyploidization ends, megakaryoblasts mature into megakaryocytes by increasing their cytoplasm through enhanced protein synthesis and by producing specific granules and a demarcation membrane system (DMS; see Glossary) (Choudry et al., 2021; Mahaut-Smith et al., 2003) (Figs 2 and 3). There is strong evidence that megakaryocyte differentiation and polyploidization are controlled by the same signalling pathways and are part of a coordinated genetic programme (Mazzi et al., 2018), but the origin and function of this polyploidization have yet to be fully elucidated.

The cell volume distribution of all cells produced by mitosis is Gaussian, as the size of the two daughter cells is approximately the size of the mother cell. However, platelets, which are not produced by mitosis, uniquely have a log Gaussian cell volume distribution with a wide dispersion (Fig. 1) (Martin et al., 2012; Trowbridge et al., 1984). The physical characteristics of cells can indicate their origin. Cell volume distribution measured by volume displacement (for example, in an electrical field using a Coulter counter or by light scattering using a flow cytometer), for cells produced by mitosis is Gaussian (a symmetrical bell-shaped curve); it has a centre of zero and a variation of unity with 95.5% of values either side of zero lying within two standard deviations. This reflects the nature of the birth of the two daughter cells from the maternal cell through an equal sharing of nuclear material and cytoplasm at cell division. This principle might be expected to apply to all circulating mammalian blood cells. However, the cell volume distribution of platelets is different and unique among blood cells. It is log Gaussian, that is, it has a low median value, followed by a mean and then a long tail to higher values with a wide dispersion (Fig. 1). A log Gaussian curve is defined as a continuous distribution of a random variable whose logarithm is normally (Gaussian) distributed. Just as the Gaussian distribution of cell volume reflects the nature of cell birth by cell division, the unique distribution of platelet volume reflects the nature of the birth of the platelet, which is not by cell division. Instead of a single cell producing two daughter cells by mitosis, a single cell (the megakaryocyte) produces on ∼3000 cells as cytoplasmic particles without any nuclei. Any valid theory of platelet production should thus be capable of explaining this unique biological distribution of cell volume. Furthermore, given that the log Gaussian distribution of platelet cell volume is unique in biology, it not only reflects a novel mechanism of cell production that is not by mitosis, but it also suggests that the mechanism was present from the origin of mammals, because all mammals have platelets and no other animals have them.

Log Gaussian distributions were first proposed to be caused by physical processes involving fragmentation by Epstein, who modelled processes used in industry (Epstein, 1948). Further analysis of the relationship between physical forces and log Gaussian distributions supported the original analysis and has wide application in industrial processes (Sommer et al., 2006). We propose that this physical principle can be used to explain the unique cell volume distribution of platelets as discussed below.

For decades, there has been much debate concerning both the site of and the mechanism of platelet production; this generated two schools of thought in the literature, each supported by evidence. One school has proposed that platelets are produced in the bone marrow, with evidence for this derived from light and electron microscopy demonstrating the anatomical proximity of cytoplasmic particles to megakaryocytes (Becker and De Bruyn, 1976; Cramer et al., 1997; Radley and Scurfield, 1980; Wright, 1910). In addition, *in vitro* studies of cultured cells supported the concept that platelets arise as protrusions from megakaryocyte cytoplasm. (Falcieri et al., 2000; Tablin et al., 1990; Topp et al., 1990). This concept was further supported by *in vivo* imaging of mouse bone marrow, which showed particles budding from megakaryocytes (Bornert et al., 2021). However, it was argued earlier using electron microscopy (Slater et al., 1983) that such protrusions might be associated with the movement of megakaryocytes out of the bone marrow into the venous circulation.

An important consideration in the interpretation of experiments where megakaryocytes are cultured *in vitro* is whether the cultured cells could produce cytoplasmic particles that were like the platelets circulating in the whole animal. For example, it has been proposed that low-ploidy megakaryocytes could produce platelets *in vitro* (Potts et al., 2020). However, the cytoplasmic particles seen by microscopy might not fulfil the definition of a platelet. Indeed, the difficulty in establishing that cytoplasmic particles seen in *in vitro* culture were indeed platelets had already been pointed out some time ago (Thiery and Bessis, 1956). A definition of characteristics needed to conclude the particles were platelets was proposed, which included them having a peripheral bundle of microtubules and functional capacity (Behnke, 1970; Martin and Gladwin, 1988). A report of diploid cultured megakaryocytes that produced platelets (Potts et al., 2020) raised the theoretical possibility that anucleate platelets evolved first, followed by the evolution of megakaryocyte polyploidy later. However, whether the cell fragments observed were actually platelets is open to interpretation. On balance, we believe that the available evidence makes it less likely that the evolution of anucleate haemostatic cells preceded the evolution of megakaryocyte polyploidy. Furthermore, for polyploidy to evolve second would offer no explanation for the platelet log Gaussian platelet cell volume distribution.

In contrast, a second school has proposed that platelets could be generated in the blood circulatory system. Circulating venous megakaryocytes have been identified in the inferior vena cava (see Glossary), the great vessel that takes blood from the bone marrow, at its various sites, to the right side of the heart and thus into the lungs via the pulmonary artery. Indeed, the numbers that were measured were sufficient to supply the daily need of platelet production (Howell and Donahue, 1937; Levine et al., 1993; Pedersen, 1971, 1978; Pedersen and Petersen, 1980; Trowbridge et al., 1984). The number of megakaryocytes entering the pulmonary circulation [6.73/ml on average (Pedersen, 1978)], if fragmented into 3000 platelets each, given an average cardiac output and knowing the average human blood volume and the platelet life time in the circulation, can be calculated as sufficient to maintain the average human blood platelet count of 200,000 per microlitre of blood for steady-state platelet production (Trowbridge et al., 1982).

Thus, it was proposed that the log Gaussian distribution of platelet volume could be explained by the fragmentation of circulating megakaryocytes in the pulmonary circulation owing to the shear forces involved (Ouzegdouh et al., 2018; Slater et al., 1983). Megakaryocytes that have travelled to the heart in relatively low flow venous blood in the inferior vena cava might enter the right ventricle of the heart where they receive the kinetic energy that accelerates them only in one possible direction – through the pulmonary artery into the capillary bed of the pulmonary circulation (Fig. 3). Compelling evidence describing pulmonary platelet production in mammals has been published (Kaufman et al., 1965; Lefrancais et al., 2017; Slater et al., 1983; Trowbridge et al., 1983; Zhao et al., 2021 preprint; Zucker-Franklin and Philipp, 2000), and the physical conditions of necessary shear force (72 dynes/cm^2^) for pulmonary platelet production in the human pulmonary circulation have been identified (Ouzegdouh et al., 2018). These shear forces are in the range that are present in the flowing blood of the pulmonary circulation. The presence of the demarcation membrane system, which occupies nearly the entire the megakaryocyte cytoplasm giving it a ‘Swiss cheese’ appearance, might aid the fragmentation of the cytoplasm into platelets (Eckly et al., 2014). Importantly, fragmentation of megakaryocytes in the pulmonary vessels of mice has been filmed in real time, and demonstrated that the origin of the fragmenting megakaryocytes was outside the lungs (Lefrancais et al., 2017). Examination of the intravital video microscopy evidence from the mouse lung presented in this study (see video 2 in the supplementary material from Lefrancais et al., 2017) shows megakaryocytes that originate from outside the pulmonary circulation fragmenting in the pulmonary circulation because of their size.

The mammalian fetus *in utero* does possess platelets but does not have a pulmonary circulation until birth. There is evidence that, during fetal life, platelet production occurs by megakaryocyte fragmentation in the capillary bed of the placenta (Woods et al., 1994). When the ductus arteriosus closes at birth and full pulmonary artery circulation is established, megakaryocyte fragmentation will switch from the placenta to the lungs (Nagasawa et al., 2016).

Finally, it is important to note that these two proposed mechanisms of platelet production are not mutually exclusive and could co-exist. The shear forces that are proposed to be involved in the formation of platelets from initial protrusions of the megakaryocyte cytoplasm into microvessels might act in the same way on the fragmenting megakaryocyte cytoplasm in microvessels of the pulmonary circulation (Junt et al., 2007). Similarly, a mechanism of platelet formation via membrane budding might also operate in the fragmenting cytoplasm in the pulmonary circulation.

**Fig. 4.**
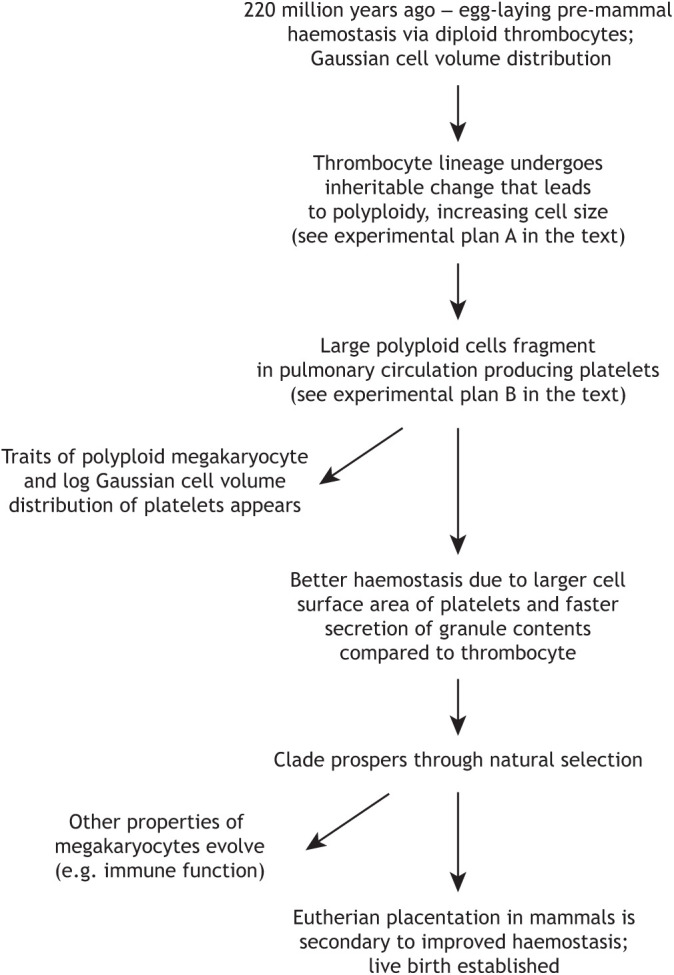
**Flow diagram illustrating the events proposed in our theory.** The diagram shows the proposed progression of events that might have led to the formation of megakaryocytes and platelets, and ultimately to mammalian placentation, as described in the text. The nature of the molecular changes that occurred to cause the initiating event in the diploid thrombocyte that led to polyploidy could be investigated using the series of experiments (experimental plan A) proposed in the section ‘Testing the hypothesis with comparative multi-omics’. A second set of experiments (experimental plan B), described in the section ‘Testing the hypothesis using cellular synthetic evolutionary biology’, could be employed to test whether the formation of large polyploid thrombocytes in birds leads to their fragmentation in the pulmonary system.

The less resolved question is what is the percentage of platelet production that takes place in the pulmonary circulation. One study proposed that a small fraction of megakaryocytes migrate to the pulmonary circulation (Dutting et al., 2017), whereas other work suggests that 50% of platelet production occurs in the lungs (Lefrancais et al., 2017). In contrast, earlier findings suggested that all platelets are produced in the pulmonary circulation (Trowbridge et al., 1982). This initial idea has credibility based on the number of megakaryocytes observed *in vivo* in the great veins that take blood to the right side of the heart, as discussed above. Our hypothesis concerns the origin of platelet production 220 million years ago; it is possible that other sites of production have evolved since then. However, we contend that it is probable that the lungs were and remain the main site of platelet production. Recent support of our proposition come from observations showing that megakaryocytes migrate to the lungs (Pariser et al., 2021). However, the authors describe parenchymal megakaryocytes in the lungs, which have an immune phenotype; these probably evolved over time in response to the local environment. Two other recent studies have also identified megakaryocyte heterogeneity (including immune competence) using single-cell analysis (Choudry et al., 2021; Sun et al., 2021). We propose that immune competence occurred in some of the megakaryocytes as evolutionary pressures were applied in mammalian development (Fig. 4). Finally, another recent study has added further evidence that platelets are produced in the lungs using an *ex vivo* lung perfusion system (Zhao et al., 2021 preprint).

## The hypothesis

Because of the uniqueness of platelets, it is reasonable to hypothesize that a unique event led to their origin. Birds and reptiles do not possess megakaryocytes or platelets; in these species, haemostasis is achieved by the aggregation of specialized diploid cells called thrombocytes. Thrombocytes mature in the bird or reptile bone marrow and then circulate, protecting the animal against blood loss at sites of injury by aggregating and secreting growth factors (Ferdous and Scott, 2015). Mammalian platelets contain three types of granules, and one of these, the alpha granule, secretes ∼100 proteins involved in mediating the haemostasis, repair and immune responses, many of which are similar to the secreted proteins of bird and reptile thrombocytes (Li et al., 2017). To perform its function of arresting bleeding and repair of injured vessels, a haemostatic cell must first attach to a site of injury of a vessel that is breached (Bizzozero, 1882). This is achieved by surface membrane receptors attaching to exposed surfaces under the damaged endothelial lining of the vessel (subendothelium or exposed vessel wall media). The attached cell then secretes, from granules in its cytoplasm, growth factors involved in repair, factors that attract other cells circulating in the blood or factors that stabilize the growing mass of cells as they become a thrombus. The greater the surface area in contact with the damaged section of the vessel, the more efficient the process of haemostasis will be. The haemostatic cell travels in the flowing blood and must be anchored to the site of bleeding by plasma membrane receptors (Ruggeri and Mendolicchio, 2007). The greater the surface area of the platelet plasma membrane, the more receptors will be presented. Also, the shorter the distance a granule in the cytoplasm of the cell must travel before it fuses with the plasma membrane, the more rapid the process of haemostasis will be. Calculations related to the change in plasma membrane surface area and speed of granule secretion are presented in Box 1. As in all modelling, the calculation makes some assumptions: (1) that the premammalian thrombocyte that was fragmented was a sphere and (2) that the particles produced from the fragmentation were spheres (modern mammalian platelets are oblate spheroids; see Glossary). However, the calculations produce such a large increase in the fragmented cell surface area and in speed of granule secretion that the assumptions of a spherical shape do not affect the magnitude of the differences. Another approximation is that each premammalian thrombocyte will fragment into 3000 particles, as modern megakaryocytes do on average. The calculations yield an 11.9-fold increase in surface area of the fragmented particles compared to the unfragmented mother cell and a 15.8-fold increase in the speed of movement of granules (Box 1). The proposed hypothesis suggests that these properties would confer an inherited advantage on the evolving clade through natural selection. Thus, platelets should be more efficient at haemostasis than a larger cell of equivalent unfragmented volume (for example the non-mammalian thrombocyte). Indeed, chicken thrombocytes are less efficient in haemostatic function compared to mouse platelets (Schmaier et al., 2011).

**Box 1. Calculation determining the increase in surface area of particles and decreased distance for secretary granules to travel following fragmentation of their mother cell**

Calculation supporting the concept that the fragmentation of a cell into 3000 particles will increase the total surface area of those particles compared to the surface area of the unfragmented cell. Furthermore, these calculations also indicate that the contents of secretary granules secreted by the particles to their outside will, on average, have less distance to travel to the plasma membrane of the fragments, compared to the distance travelled in the unfragmented cell, thereby, on average, increasing the speed of secretion. The hypothesis argues that this fragmentation gave the animal an advantage in arresting bleeding.

Assume *C* is a cell, which is assumed to be a perfect sphere of radius *r*. The volume of *C* is then 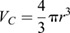, and its surface area is *A*_*C*_=4π*r*^2^. If the volume of the nucleus is *V*_*C*_/4, then the volume of the cell excluding the nucleus is 
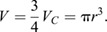
 If the cell, excluding the nucleus, is broken into 3000 identical spheres, each sphere has volume 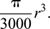 Suppose *S* is one of these spheres, which is of radius *r*_*S*_ and volume 
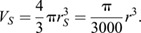
 It follows that 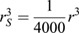, meaning 
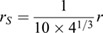
.

Numerically, 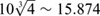, to three decimal places, so 
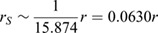
. From this, we calculate the surface area of *S* to be 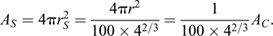

Since *S* is arbitrary, one of 3000 spheres, it can be deduced that the total surface area of the three thousand spheres is 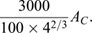

Numerically, this is approximately 11.906*A*_*C*_, to three decimal places.

Suppose *X* is a point in *C* which is being ejected radially outwards from the centre of *C* at constant velocity *v*. Since the radius of *C* is *r*, the time taken for *X* to travel from the centre of *C* to its surface is 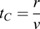. Suppose *S* is one of the 3000 identical spheres from above, whose radius is *r*_*S*_, and suppose *Y* is a point in *S* that is also being ejected radially outwards from the centre of *S*, also at velocity *v*. Then the time take for *Y* to travel from the centre to the surface of *S* is 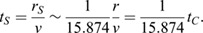

Therefore, we would like to propose that the mammalian megakaryocyte represents an evolutionary development from a nucleated diploid cell, analogous to the thrombocyte of modern reptiles and birds, through an event that occurred in a single animal, most likely a precursor of the modern order of monotremes (which possess platelets and megakaryocytes) at ∼220 million years ago (Meredith et al., 2011). We hypothesize that an inheritable modification occurred specifically in the genetic programme of the haemostatic cell lineage of this founder animal, which altered the control of cell division causing polyploidization. This inheritable change, with possibly trans-generational epigenetic (see Glossary) components (Ashe et al., 2021; Lind and Spagopoulou, 2018), altered the expression and/or function of a mitotic component and resulted in the acquisition of polyploidy in a precursor cell that would have otherwise become a diploid haemostatic cell. These larger cells, proportionate to their level of polyploidy, would then have circulated after leaving the bone marrow. Before the change that caused polyploidy, the original haemostatic cells (thrombocytes) would have passed, unfragmented, through the capillary bed of the pulmonary circulation, the first capillary bed encountered after the cells left the bone marrow. Because of the larger size of the polyploid cells (the future mammalian megakaryocytes), they would have become fragmented into cytoplasmic particles owing to the presence of the appropriate physical and biochemical conditions (Ouzegdouh et al., 2018), as observed in modern mouse pulmonary vessels (Lefrancais et al., 2017). The haemostatic response to a broken vessel wall is more effective owing to the larger surface area of plasma membrane in contact with the damaged vessel area. Furthermore, the effectiveness of the haemostatic response is also related to the speed of secretion of the contents of platelet alpha granules, which contain haemostatic and reparative proteins. Because of the smaller size of the fragments, the distance from a granule therein to the plasma membrane is on average shorter, making the secretory response more rapid. These fragments (the future mammalian platelets) were thus more haemostatically effective compared to the original single non-polyploid haemostatic cell of similar combined volume that the animal had (Box 1). Therefore, owing to the advantage of being able to arrest bleeding more effectively, the progeny of the animal would have had an advantage through natural selection. This inheritable trait would have been passed on to the offspring of the founder, which would have thrived and expanded. This event would have been a key step in the evolution of mammals, and this more effective haemostasis system could have also been key for the evolution of the placenta (Martin and Wagner, 2019).

The live-imaging data referred to above (Lefrancais et al., 2017) illustrates what we proposed happened immediately after the generation of large cells, following the *de novo* occurrence of polyploidy in the thrombocyte precursor of the animal. These observations indicate that the megakaryocyte is clearly too large to pass through the vessels of the lungs, which decrease in size as they carry blood to the alveolar capillaries for oxygenation (Lefrancais et al., 2017). Indeed, megakaryocytes can be seen splitting at the first bifurcation of the vessels, before the two halves then further break up at the next bifurcations (Lefrancais et al., 2017). This fragmentation, caused by physical forces provided by the kinetic energy of the right ventricle, could give rise to a collection of cytoplasmic particles that follows a log Gaussian cell volume distribution, as predicted (Epstein, 1948; Sommer et al., 2006) and as observed in the volume distribution of modern mammalian circulating platelets (Fig. 1).

We would also like to suggest that the polyploid megakaryocyte ancestor would have then, likely through further adaptive evolutionary events, acquired the same properties that are now found in the mammalian megakaryocyte, including a larger membranous system compared to its diploid progenitor, giving rise to a DMS of complex invaginations, which aids cytoplasmic fragmentation. Such further evolutionary events might have occurred because of the increase in gene expression associated with an increased number of chromosomes in the cell. Thus, we suggest that the increased protein production, which is necessary to produce the DMS and the granules observed in mature megakaryocytes, occurred during evolution, thanks also to the increased number of genes due to polyploidy (Choudry et al., 2021). This hypothesis is consistent with the observation that, during differentiation, polyploidy occurs before the final maturation of the megakaryocyte (Fig. 2). Although physical forces are involved in megakaryocyte fragmentation (Ouzegdouh et al., 2018), cellular biochemical processes are involved in the fusion of membranes to produce the final cytoplasmic oblate spheroid, which is the platelet. We propose that the biological changes, referred to above, of platelets budding from megakaryocytes and the formation of proplatelets, which can be observed in some *in vitro* and *in vivo* experiments, might occur during fragmentation of cytoplasmic particles in the pulmonary circulation. Notably, this concept would unify the two schools of thought in the literature regarding the mechanism and site of platelet production.

### Testing the hypothesis with comparative multi-omics

The evidence that polyploidization is an intrinsic component of megakaryocyte differentiation indicates that these two events are regulated by a coordinated and specific gene regulatory programme (Mazzi et al., 2018). Part of this programme must involve one or more alterations in the expression of mitotic factor(s) that result in polyploidization, because studies have indicated that megakaryocyte polyploidization is caused by abortive mitoses known as endomitoses (Nagata et al., 1997; Vitrat et al., 1998). The exact nature of the mitotic defect(s) and their underlying molecular mechanism(s) are still unclear. It has been debated whether megakaryocyte polyploidization resulted from a failure in chromosome segregation (karyokinesis) or in the separation of the two daughter cells at the end of mitosis (cytokinesis). However, several studies strongly indicate that cytokinesis failure is the main origin of polyploidy in megakaryocytes (Geddis et al., 2007; Lordier et al., 2008), including the observation of multinucleate cells (which is the expected outcome of cytokinesis failure) in megakaryocytes that are derived from cord blood or differentiated *in vitro* from human pluripotent stem cells (Moreau et al., 2016). However, mature highly polyploid megakaryocytes present polylobate nuclei rather that multiple nuclei (Fig. 3), indicating that a combination of both karyokinesis and cytokinesis defects is the most likely cause of polyploidy in these cells (Lordier et al., 2012b; Mazzi et al., 2018). Together, this evidence indicates that cytokinesis failure could initially cause the formation of tetraploid (4N) megakaryoblasts, which could then increase their ploidy through subsequent endomitotic events that are characterized by both defects in chromosome segregation and cytokinesis, which are both typically observed in polyploid cells (Fig. 2).

It has been suggested that silencing of the non-muscle myosin IIB heavy chain (MYH10) during megakaryocyte differentiation might contribute to polyploidization, as reduced myosin activity could alter the dynamics of the actomyosin ring that divides the two daughter cells during cytokinesis (Lordier et al., 2012a). However, this is unlikely to be the only factor for two main reasons. First, defects in actomyosin ring constriction would cause cells to fail cytokinesis early, during the initial ingression of the cleavage furrow (D'Avino et al., 2015). This contrasts with the observation of late cytokinesis failure (i.e. after completion of furrow ingression) in primary human megakaryocytes (Geddis et al., 2007; Lordier et al., 2008). Second, defects in actomyosin ring constriction would not directly affect karyokinesis, although polyploid cells are more likely to display abnormal chromosome alignment and segregation (Storchova and Pellman, 2004). The presence of lagging chromatin and subsequent cleavage furrow regression observed during cytokinesis in cultured megakaryocytes (Lordier et al., 2012b) suggests that cytokinesis failure could be caused by the inability of the daughter cells to physically separate (a process known as abscission) because of the presence of DNA at the cleavage site. Recent studies have revealed that the chromosomal passenger complex (CPC) is the key factor that senses the presence of DNA at the cleavage furrow in order to prevent abscission and the formation of two genetically abnormal daughter cells (D'Avino and Capalbo, 2016). The CPC is one of the major regulators of cell division in all eukaryotes and is composed of four subunits – the scaffolding component inner centromeric protein (INCENP), borealin, survivin (also known as BIRC5) and Aurora B kinase (AURKB) (Carmena et al., 2012). The crucial role of the CPC in controlling abscission makes it a strong candidate for being one of the mitotic factors altered or inhibited during the process of megakaryocyte polyploidization. However, previous studies of the possible role of the CPC in megakaryocyte polyploidization have led to conflicting results. Initial studies indicated that AURKB expression was downregulated during human megakaryocyte differentiation induced *in vitro* (Kawasaki et al., 2001), and both AURKB and survivin were found to be mis-localized and reduced, or even absent, during endomitosis in mouse bone marrow megakaryocytes (Zhang et al., 2004). However, these findings were subsequentially contradicted by another study showing that AURKB and survivin were normally expressed and localized in cultured human megakaryocytes (Lordier et al., 2010). Moreover, AURKB inhibition or deletion did not impair megakaryocyte cytokinesis and polyploidization (Lordier et al., 2010; Trakala et al., 2015), although AURKB inhibition affected chromosome segregation (Lordier et al., 2010). These results were interpreted to indicate that the CPC is dispensable for cytokinesis and polyploidization during megakaryocyte differentiation. However, as the CPC is a key regulator of cytokinesis and abscission in all other cell types (D'Avino and Capalbo, 2015), these results could also indicate that the functions of the CPC might be specifically inactivated during megakaryocyte cytokinesis in order to induce polyploidy. This could be achieved through different mechanisms, including for example post-translational modification of one or more CPC component, inhibition of the association of the CPC with one or more cytokinesis partners [such as the kinesin KIF20A (also known as MKLP2)], or the inability of the CPC to regulate some of its targets during cytokinesis. In our opinion, all these possibilities, which are compatible with the published evidence, deserve further investigation.

As an increase in ploidy is achieved through repeated cycles of endomitosis, it is evident that, regardless of the nature of the alterations in the cell division factors that are responsible for megakaryocyte polyploidization, these alterations must be transmitted from the mother cell to the daughters through an inheritable, most likely epigenetic, mechanism. Therefore, a possible unbiased experimental approach to identify such alterations could be a comparative multi-omics analysis of the epigenome, transcriptome and proteome of megakaryoblasts differentiated *in vitro* with a different ploidy status (Raslova et al., 2007), and to compare these results with similar multi-omics analyses of bird thrombocytes (Ferdous et al., 2016) (Fig. 4, experimental plan A). Although these experiments might not be able to exactly pinpoint the change that occurred 220 million years ago in the mammalian ancestor, it is very likely that they might help to identify specific differences in the genetic programmes of thrombocytes compared to megakaryocytes, such as to indicate which factor(s) might have been involved in the event that led to the initial polyploidization. Subsequent targeted cell biology experiments could then unequivocally determine the origin(s) and mechanism(s) that lead to polyploidy during megakaryocyte differentiation (Fig. 2). These could include analysis of mitotic protein expression and distribution during mitosis by western blotting, time-lapse imaging and immunofluorescence analysis, as well as trying to recapitulate the loss or inactivation of specific mitotic factors by gene knockdown and/or knockout.

### Testing the hypothesis using cellular synthetic evolutionary biology

Another experimental objective is to cause polyploidy in the thrombocyte precursor cells in an animal that does not have megakaryocytes or platelets, thus recapitulating the initial event proposed in our theory (Fig. 4, experimental plan B). The chicken is a good model of pre-mammalian haemostasis because, like all birds and reptiles, haemostasis in chickens is mediated by circulating thrombocytes with diploid (2N) nuclei. *In vivo* experiments can be devised to produce a ploidy increase in dividing bone marrow thrombocyte precursors, producing circulating high-ploidy thrombocytes. If our theory is correct, these will produce platelets in chicken lungs as is the case in mammals. Another advantage of using chickens is that they have vessels from which it is easy to take blood. Although there are some anatomical differences between bird and mammalian lungs, blood flow through the pulmonary capillary bed is essentially the same; however, the avian capillaries are more rigid (Watson et al., 2008). There are two agents with an appropriate plasma half-life and tissue IC_50_, that can be given though the intravenous route to induce polyploidy. Firstly, the potent selective AURKB inhibitor barasertib (AZD1152) (Wilkinson et al., 2007) is expected to cause polyploid thrombocytes, owing to the role of AURKB as an important mitotic regulator and thus a strong candidate for involvement in megakaryocyte polyploidization (see above). Secondly, the selective Rho kinase (ROCK) inhibitor fasudil (HA-1077) (Abe et al., 2014) might be used because ROCK phosphorylation of the myosin regulatory light chain is essential for cytokinesis, and there is published evidence that ROCK inhibition increases the ploidy of megakaryocytes (Lordier et al., 2008). Such experiments would only need to take place over only a few days and therefore it is not envisaged that these agents will have a significant adverse systemic effect. The presence of any polyploid precursors cells of thrombocytes can be assessed by propidium iodine staining and flow cytometry. However, blood samples for these studies would need to be taken from the inferior vena cava, before the blood reaches the pulmonary circulation. Moreover, if the hypothesis is correct, blood taken from the peripheral circulation would be expected to show the presence of anucleate cytoplasmic particles with a log Gaussian particle volume distribution, just as mammalian platelets, and any anatomical characteristics of mammalian-like platelets in the chicken could be assessed by electron microscopy, such as the presence of a peripheral bundle of microtubules necessary for platelet secretion and the presence of cytoplasmic granules. Furthermore, if platelets are indeed produced, functional studies could be performed to demonstrate whether secretion occurs in response to the platelet receptor agonists ADP, collagen and thrombin, as would be expected from bona fide platelets.

## Conclusions

We propose here a new evolutionary mechanism, a single biological event, that could aid our understanding of evolution. Previous theories of evolution have not been supported by controlled interventional experimentation. The results of the experiments proposed here could explain the origin of platelets at the biological level and therefore help to identify the initial conditions for the evolution of all mammals (via the development of the placenta), including humans. Such studies are able to provide support and an experimental explanation for the role of polyploidy in mammalian evolution. Polyploidy can offer several evolutionary advantages in coping with stress and environmental changes, owing to the presence of multiple sets of chromosomes (Van de Peer et al., 2017). The occurrence of polyploidy appears to be widespread in plant evolution compared to a much rarer number of polyploidy events documented in animals (Van de Peer et al., 2017), although recent evidence suggests that polypody in animal could have been underestimated (Peterson and Fox, 2021). One of the possible reasons for this difference could be the presence of centrosomes in animal cells. Indeed, the presence of multiple centrosomes in polyploid animal cells causes problems in the alignment and segregation of the chromosomes during cell division, which can generate genomic instability (Cosenza and Kramer, 2016; Duncan et al., 2010; Schoenfelder et al., 2014). If indeed proven, our theory would provide, to our knowledge, the only example of a polyploidy event that is important and beneficial for mammalian evolution. As outlined here, experiments aimed to support our theory could include synthetic evolutionary biology at the cellular level in order to recreate an evolutionary event that happened about 220 million years ago. Synthetic evolutionary biology has been attempted at the protein level before, in particular the engineering of the components of cells (Castle et al., 2021), but we are unaware of synthetic evolutionary biology experiments at the cellular level.

The occurrence of a evolutionary change that occurred at a moment might have taken place in other situations or may do so in the future. We hope that the ideas presented here will give rise to debate, particularly about the potential speed of evolution in particular situations.

Furthermore, the multi-omics and cell biology experiments we propose might not only identify the events responsible for megakaryocyte polyploidization, but also reveal how these cells are able to bypass and/or overcome the surveillance mechanisms that prevent polyploidization in the vast majority of human tissues (Ganem and Pellman, 2007).

Finally, the haemostatic properties of the platelet, which arose 220 million years ago, were needed for the success of live birth by stopping bleeding at the separation of the placenta (Martin and Wagner, 2019). Nevertheless, those same properties have become prothrombotic during the inappropriate aggregation of platelets in modern humans, leading to vascular thrombosis in heart attack and stroke (Martin et al., 2012). The single archaic change causing polyploidy thus had profound physiological and pathological consequences.
